# Treating Target Behaviors of Autistic Individuals With Applied Behavior Analysis: An Ongoing Replication Study

**DOI:** 10.7759/cureus.54109

**Published:** 2024-02-13

**Authors:** Tami Peterson, Jessica Dodson, Frederick Strale,

**Affiliations:** 1 Hyperbaric Oxygen Therapy, The Oxford Center, Brighton, USA; 2 Applied Behavior Analysis, The Oxford Center, Brighton, USA; 3 Biostatistics, The Oxford Center, Brighton, USA

**Keywords:** autism spectrum disorder (asd), target behaviors, repeated measures design, naturalistic environment training, mass trials, discrete trial training, applied behavior analysis

## Abstract

Background

Ongoing outcome data replication on target behaviors with autistic individuals using applied behavior analysis (ABA) confirms its effectiveness and remains an essential evidenced-based standard of care. This replication study aims to further confirm the impact of discrete trial training and mass trials on general target behaviors within a naturalistic environment.

Methods

Data was gathered from 92 children and four adult autistic individuals over one month from 7/7/23 to 8/8/23 using a repeated measures design. This study used a retrospective chart review with general target behaviors to determine the effectiveness of ABA treatments using discrete trial training and mass trials across time and age categories in a naturalistic environment.

Results

A mixed analysis of variance (ANOVA) indicated statistical significance (sphericity assumed), F(2,168) = 31.663, p < 0.001 (time). Multiple comparisons using bootstrapped paired t-tests indicated p < 0.001 on the three comparisons. There was a significant interaction effect (sphericity assumed) with time x age category, F(8,168) = 2.918, p = 0.004. Interaction contrasts indicated statistically significant differences over time within the 1-4 years, 5-8 years, and a portion of 9-12 years, and not within the 13-16 years and 17-73 years age groups.

Conclusions

Autistic individuals receiving ABA demonstrated statistically significant improvement in target behaviors over one month. There was a significant interaction between time and age on target behaviors, suggesting a significant association between time and age categories. The reporting of ongoing intervention outcomes provides further justification for continued treatments relative to target behavior mastery with autistic individuals.

## Introduction

Background

Autism spectrum disorder (ASD) is a neurodevelopmental disorder that impacts social communication and includes repetitive patterns of behavior and possible fixed and restricted interests. Applied behavior analysis (ABA) is an effective and evidence-based treatment for ASD symptoms. ASD prevalence is one in 36 individuals as of 2023, with a male-to-female ratio of 4:1, and is characterized by delay and difficulty in social communication and behavior. Prevalence estimates have increased, indicating a wide variation among different ethnicities and socioeconomic backgrounds. Children with ASD undergo many interventions at home, in the clinic, and at school to help with issues inherent to the disorder [1-3].

Behavioral interventions based on ABA form current evidence-based practices in treating ASD. Research is scarce relative to the broad effects of intensive, repetitive, discrete trial training and mass trials combined with a naturalistic environment with large N designs, as measured by overall general target behaviors. Does ABA training with autistic individuals in discrete trial training, mass trials, and naturalistic environment training significantly affect the cohorts' ability to achieve target behaviors over time?

Ongoing reporting of intervention outcome data relative to target behaviors in the treatment of autistic individuals with ABA is necessary to confirm its effectiveness further. Despite the variability in efficacy in the clinical implementation of ABA due to increasing numbers of individuals diagnosed with ASD, confirmation of ABA as an evidence-based standard of care for ASD with new ongoing data remains essential [4,5].

Original studies

Peterson, Dodson, Hisey, Sherwin, and Strale [6] and Peterson, Dodson, and Strale [7] discussed ABA efficacy in general, using large N designs (n = 100 and n = 98) with mixed models in a naturalistic environment and measured target behaviors relative to the impact of discrete trial training and mass trials on target behaviors in individuals with autism with repeated measures. Results indicated that ABA significantly increased general aggregate target behaviors over seven time points covering three months [6] and in a replication study over three time points covering one month [7]. Both studies observed statistically significant increases in mean and median measurements of the number of multiple raters composite general target behaviors achieved per session. The multiple comparisons between time points in both studies indicated an upward trend of improvement and statistically significant differences between time points with medium to large effect sizes [6,7]. Significant age effects were discovered, the most prominent being in the 13-16 years age category [6], with a non-significant age effect found in the first replication study [7].

Purpose of replication

This replication study’s primary objective is to determine the effectiveness of ABA treatment in a retrospective cohort of n = 103 autistic individuals administered ABA with functional analysis over three time points covering one month between 7/7/23 and 8/8/23. The participant cohorts treated with ABA are hypothesized to demonstrate statistically significant progress toward target behavioral goals.

The secondary objective is ascertaining whether an association exists between the three time points and age categories. It is hypothesized that the participant cohorts treated with ABA will demonstrate statistically significant progress toward target behavioral goals. The independent variable of time will significantly interact with age categories to produce significant effects between time within age categories.

## Materials and methods

Participants and setting

Participant cohort data (n = 103) was gathered using a retrospective chart review from the “Catalyst” tracking software (DataFinch Technologies, Atlanta, USA) of individuals who received ABA treatment. A repeated measures analysis covered three time points (baseline, two weeks, and four weeks) between 7/7/23 and 8/8/23, measuring cumulative target behaviors. Reporting and manuscript preparation adhered to Strengthening the Reporting of Observational Studies in Epidemiology (STROBE) guidelines.

Method of data collection and dependent variables with operational definitions

The dependent variable was the number of cumulative target mastery behaviors achieved per session, measured at three time points: Time 1 (baseline), Time 2 (two weeks), and Time 3 (four weeks). “Catalyst” is an ABA data collection software that produces automated progress notes for repeated measures outcome data for discrete trial teaching targets with frequency and rate data. Graphs in Catalyst track numerical progress and lack of numerical progress with targeted behaviors and automatically determine mastered targets as respective criteria are achieved.

Experimental design - repeated measures over time 

Observation and analysis of the empirical effectiveness of treatments are more precise in repeated measures designs because they allow researchers to measure how the treatment affects each individual. Repeated measures designs look at response outcomes measured on the same experimental unit at various times or under different conditions. In repeated measures designs, each subject serves as its own control [8].

Interobserver reliability

A two-way random effects model was computed where people's effects and measures effects are also random. We used the intraclass correlation coefficient, ICC (2), which is used when multiple measurements are made from each averaged rater. The ICC (2) value was 0.956 (95% CI: 0.931-0.972), indicating excellent agreement between the raters. This value was more significant than the average Pearson r (0.956), suggesting that the ICC (2) was equally sensitive to the variability among raters and measurements. Cronbach’s alpha for the three time point variables was r = 0.974 [9,10].

Power analysis - study size

A retrospective power analysis was conducted using G*Power 3.1 (Heinrich-Heine-Universität Düsseldorf, Düsseldorf, Germany) [11] and indicated that a total sample size of n = 27 participants would be required to demonstrate a high group effect size (0.80) for a repeated measures design with nominal alpha (α) = 0.05 using a (between x within) repeated measures mixed analysis of variance (ANOVA), with a power equal to 0.987. Given the analysis parameters, there is a high likelihood that this current retrospective trial with n = 103 participants indicated an acceptable sample size criterion.

Statistical methods

Statistical Package for the Social Sciences (IBM SPSS Statistics for Windows, IBM Corp., Version 29.0, Armonk, NY) was used for all descriptive and inferential analyses. The nominal alpha (α) was set at 0.05. If p-values were less than 0.05 (p < 0.05), the null hypothesis was rejected and statistical significance was inferred. Demographics and baseline characteristics were summarized for all 103 subjects. Summary statistics for categorical variables, gender, and race/ethnicity, and for continuous variables, age, Time 1, Time 2, and Time 3 (mean, standard deviation, median, and range) were generated.

A mixed (between x within) ANOVA was used to determine the overall statistical significance between the three (Time 1 to Time 3) levels of the independent variable, as well as any interaction effects between the fixed factor (age category) and the three repeated measures time points assessing target behaviors [12].

If an overall significant omnibus F statistic was detected (p < 0.05) within the mixed (between x within) ANOVA, a step-down analysis was performed using resampling multiple comparison procedures in the form of bootstrapped paired tests (1000 replications). Using bootstrapping with paired t-tests, resampling methods mitigate potential multiplicity, thereby reducing familywise error rate (FEW) likelihoods [13].

The Bonferroni correction was also used as α = 0.05/3 = 0.017. Therefore, with these multiple comparisons, if p < 0.017, the null hypothesis was rejected and statistical significance was inferred [14].

If an overall significant omnibus interaction F statistic was detected (p < 0.05) within the mixed (between x within) ANOVA, a step-down analysis was performed using interaction contrasts comparing each between-subjects factor with the within-subjects factor to determine specifically where the significant differences (effects) came about.

Institutional review board approval

Consent was obtained or waived by all participants in this study. The Oxford Center was issued approval number 1-1703366-1 from the WIRB-Copernicus Group Institutional Review Board (WCG IRB). The authors declare that this research investigation involves minimal risk and complies with the Belmont Report Regulations 45 CFR 46 2018 Requirements (2018 Common Rule) Section 46 Subpart A Basic HHS Policy for Protection of Human Research Subjects, 46.104 Exempt Research Paragraph d (1), (2), and (2) (ii) and 46.117 Documentation of Informed Consent Paragraph c (1) (ii). This study also conformed to the guidelines outlined in the 1964 Declaration of Helsinki.

## Results

Descriptive statistics

For the sample of 103 autistic individuals, the age was (M = 9.23, SD = 7.94), the median was 8, the minimum was 2, and the maximum was 73. There were seven missing values. There were 75 males (72.8%) and 27 females (26.2%), with one missing value. There were 75 Caucasians (72.8%), six Asians (5.8%), three Hispanics (2.9%), 12 Middle Eastern (11.7%), and seven African Americans (6.8%). There were no missing values. In terms of age categories, 18 (17.5%) were in the 1-4 years category, 37 (35.9%) were in the 5-8 years category, 22 (21.4%) were in the 9-12 years category, 15 (14.6%) were in the 13-16 years category, and four (3.9%) were in the 17-73 years category. There were seven (6.8%) missing values. Four subjects were over 17 years old, e.g., 18 years old, 20 years old, 26 years old, and 73 years old. Table 1 displays the results of descriptive statistics for repeated measurements by age group categories.

**Table 1:** Descriptive statistics for repeated measurement by age group

Outcome Variable	Age Category	Mean	Std. Deviation	N
Targets Mastered Baseline	1-4 yr	23.5625	20.24177	16
	5-8 yr	16.9714	12.93533	35
	9-12 yr	11.7727	12.79111	22
	13-16 yr	23.9167	29.41075	12
	17-73 yr	14.0000	8.52447	4
	Total	17.6742	17.47191	89
Targets Mastered Two Weeks	1-4 yr	30.0625	25.71502	16
	5-8 yr	21.8286	16.19819	35
	9-12 yr	15.1364	15.58783	22
	13-16 yr	25.5833	33.09616	12
	17-73 yr	15.2500	10.24288	4
	Total	21.8652	20.97574	89
Targets Mastered Four Weeks	1-4 yr	38.6250	31.72355	16
	5-8 yr	29.4000	19.98705	35
	9-12 yr	17.5455	18.17567	22
	13-16 yr	29.0000	36.56874	12
	17-73 yr	18.7500	16.52019	4
	Total	27.5955	25.17291	89

The results of descriptive statistics relative to Time 1, Time 2, and Time 3 measurements are presented in Table 2. An increase in means and medians over the three time periods is noted.

**Table 2:** Descriptive statistics for repeated measurement

Descriptive statistics		Time 1	Time 2	Time 3
N	Valid	95	95	95
	Missing	8	8	8
Mean		17.1263	21.3895	26.9684
Median		11	16	17
Std. Deviation		17.15712	20.56091	24.6367
Minimum		0	1	1
Maximum		101	116	128

Inferential statistics

Mixed (between x within) ANOVAs were performed with accompanying post hoc analyses and interaction contrasts, beginning with an analysis of the underlying assumptions. The three time point measurements are scale variables and continuous variables (ratio/interval). The within-subjects factor consists of the same subjects measured at three time points.

The between-subjects factor consists of the age category. There were four outliers in Time 1 (Case #s 38, 43, 55, and 99), five outliers in Time 2 (Case #s 13, 38, 43, 55, and 99), and four outliers in Time 3 (Case #s 38, 43, 55, and 99). Because of the nature of the learning progress of the population of autistic children and this repeated measures analysis, the outliers were retained as they are natural to the study’s research question.

The timepoint variables demonstrated a non-normal configuration. Q-Q plots were non-aligned. Also, the skewness scores for all three time points were outside the typically accepted range of -1 to +1 (M = +1.75, SD = 0.247). Mixed (between x within) ANOVA is quite "robust" to violations of normality, meaning that the assumption can be somewhat violated and still provide valid results [15].

Homogeneity of variances for each combination of the within-subjects factor and between-subjects factor is required. “Sphericity” relates to the variances of the differences between the related groups of the within-subjects factor for all groups of the between-subjects factor (the within-subjects factor and the between-subjects factor) must be approximately equal.

Mauchly’s test of sphericity was significant. This indicates that the assumption of sphericity was not met; Mauchly’s W = 0.455, Approximate Chi-Square = 65.351, df = 2, p < 0.001, Greenhouse-Geyser Epsilon = 0.647, Huynh-Feldt Epsilon = 0.684, Lower-bound = 0.500. Therefore, the variances of the differences among the combinations of related groups are not equal. Consequently, Greenhouse-Geyser Epsilon was used to adjust the degrees of freedom for the averaged tests of significance [12]. Corrected tests (F-values) are reported in the Results section.

Also, Box's M was significant with M = 80.412, F(24,862) = 2.753, p < 0.001, indicating heterogeneity of variance and covariances between covariance matrices.

Several investigations [12,15] and others using Monte Carlo Simulations to estimate the robustness of generalized linear models (GLMs), of which mixed (between x within) ANOVA is a member, have been reported, suggesting robustness (the likelihood of Type I errors is reduced).

Mixed ANOVA (Between x Within) - Main Effects 

There was a significant main effect (sphericity assumed) on the dependent variable (targets mastered) across time, F(2,168) = 31.663, p < 0.001), indicating an overall statistically significant effect (increase in targets mastered) detected across the three time points of the independent variable (time) over one month.

Post Hoc Analyses 

Post hoc analysis was conducted using bootstrapped (1000 replications) paired t-tests for multiple comparisons with a Bonferroni corrected (0.05/3) α = 0.017. Results indicated statistical significance for all three time point comparisons (Time 1 vs Time 2; t(94) = -7.142, p < 0.001), (Time 1 vs Time 3; t(94) = -8.984, p < 0.001), and (Time 2 vs Time 3; t(94) = -4.114, p < 0.001); across the time dimension, all p-values were < 0.05 and were statistically significant at α = 0.05.

Mixed ANOVA - Interaction Effects - Time x Age Category 

There was a significant interaction effect (sphericity assumed) on the dependent variable (targets mastered) across time and age category, F(8,168) = 2.918, p = 0.004), indicating a statistically significant interaction effect (there is an association) detected across the three time points of the independent variable (time) with age category.

Interaction Contrasts

Tests of simple main effects were conducted. The results are presented in Table 3.

**Table 3:** Interaction contrasts - time x age category comparisons with Time 1, Time 2, and Time 3 target behaviors *: Based on estimated marginal means ^b^: Statistical significance p < 0.017, adjustment for multiple comparisons per Bonferroni

Age Category	(I) Time	(J) Time	Mean Difference	Std. Error	p^b^	95% Confidence Interval for Difference^b^	
						Lower Bound	Upper Bound
1-4 yr	1	2	-6.500^*^	1.388	< 0.001	-9.892	-3.108
		3	-15.062^*^	2.602	< 0.001	-21.419	-8.706
	2	1	6.500^*^	1.388	< 0.001	3.108	9.892
		3	-8.563^*^	1.770	< 0.001	-12.887	-4.238
	3	1	15.063^*^	2.602	< 0.001	8.706	21.419
		2	8.563^*^	1.770	< 0.001	4.238	12.887
5-8 yr	1	2	-4.857^*^	0.939	< 0.001	-7.150	-2.564
		3	-12.429^*^	1.759	< 0.001	-16.727	-8.131
	2	1	4.857^*^	0.939	< 0.001	2.564	7.150
		3	-7.571^*^	1.197	< 0.001	-10.495	-4.648
	3	1	12.429^*^	1.759	< 0.001	8.131	16.727
		2	7.571^*^	1.197	< 0.001	4.648	10.495
9-12 yr	1	2	-3.364^*^	1.184	0.017	-6.256	-0.471
		3	-5.773^*^	2.219	0.033	-11.194	-0.352
	2	1	3.364^*^	1.184	0.017	0.471	6.256
		3	-2.409	1.510	0.343	-6.097	1.279
	3	1	5.773^*^	2.219	0.033	0.352	11.194
		2	2.409	1.510	0.343	-1.279	6.097
13-16 yr	1	2	-1.667	1.603	0.905	-5.583	2.250
		3	-5.083	3.005	0.283	-12.424	2.257
	2	1	1.667	1.603	0.905	-2.250	5.583
		3	-3.417	2.044	0.295	-8.410	1.577
	3	1	5.083	3.005	0.283	-2.257	12.424
		2	3.417	2.044	0.295	-1.577	8.410
17-73 yr	1	2	-1.250	2.777	1.000	-8.033	5.533
		3	-4.750	5.204	1.000	-17.464	7.964
	2	1	1.250	2.777	1.000	-5.533	8.033
		3	-3.500	3.540	0.977	-12.149	5.149
	3	1	4.750	5.204	1.000	-7.964	17.464
		2	3.500	3.540	0.977	-5.149	12.149

## Discussion

Summary of findings

This study used discrete trial training, an applied behavior analytic intervention that simplifies complexity. Discrete trial training takes large, gross tasks and reduces them to small, individualized tasks. It teaches autistic individuals with straightforward and systematic methods. Mass trials are a method within discrete trial training that repeatedly presents the same stimulus until a correct response is achieved. This is all accomplished within a naturalistic environment that teaches behavioral skills within the natural context of a learning environment. The respective learner’s individual preferences and partialness serve as the motivation. The effects of a mixed model of discrete trial training, mass trials, and naturalistic environment training in autistic children are noteworthy as they can assist with various aspects of learner's cognitive, language, social, and adaptive skills development. The benefits of discrete trial training include helping autistic children learn appropriate responses to different situations, which can enhance communication, their relationships with family, classmates, and peers, and overall quality of life. Acquisition of skills such as matching, discrimination, and imitation using this form of ABA can enhance learning that is difficult to acquire in naturalistic settings [6,7,16-24]. 

Mass trials assist autistic children with acquiring new behaviors more quickly and efficiently as exposure to the same or similar stimulus increases. This ABA method can help increase and retain learned behaviors over time by strengthening memory and improving recall abilities. Naturalistic environment training assists autistic children with generalization skills transferred from discrete trial training to different contexts, including people, materials, and settings. Naturalistic environment training also helps with increased motivation, spontaneity, and engagement by utilizing reinforcements that occur naturally and are aligned with learner interests [6,7,16-24]. 

This study’s primary objective was to replicate new data on three time points between 7/7/23 and 8/8/23, confirming the effectiveness of ABA treatment in a retrospective cohort chart review of n = 103 autistic children treated with ABA, with functional analysis over three time points covering one month. The statistical results suggested that ABA intervention over three time point measurements significantly increased target behaviors. Specifically, the multiple comparisons between each time point indicated an upward trend of improvement and statistically significant differences between time points. This hypothesis was confirmed. 

Our secondary objective was to determine if an association existed between the three time points and age categories. It was hypothesized that the child cohorts treated with ABA would demonstrate statistically significant progress toward target behavioral goals. The independent variable of time would significantly interact with age categories to produce overall significant effects between time within age categories. This hypothesis was confirmed within the 1-4 years, 5-8 years, and 9-12 years age groups (p < 0.05) and not confirmed within the 13-16 years and 17-73 years age groups (p > 0.05). Our study found that autistic children receiving ABA significantly improved target behaviors over one month. Also, a significant interaction was discovered between the independent variable (time) and the age category on target behaviors, suggesting a significant association between time and age categories. 

Comparison with the original study

This study found results similar to those of Peterson, Dodson, Hisey, Sherwin, and Strale [6] and Peterson, Dodson, and Strale [7] with statistically significant findings relative to the efficacy of ABA treatments in a retrospective cohort snapshot of 103 autistic children. As with the first two studies, functional analysis consisting of discrete trial training and mass trials was utilized within a naturalistic environment. Unlike the second study [7], we found a statistically significant interaction (time x age) within many age groups, as mentioned above.

Implications

This study showed evidence to increase confidence in our first two studies' results [6,7]. The mixed model consisting of discrete trial training and mass trials within a naturalistic environment with autistic individuals strengthens the development of cognitive, language, social, and adaptive behaviors. The increase in this replication study with general target mastery behaviors over the designated three time points covering one month is noteworthy. Ongoing studies of general ABA broad effectiveness, namely, with discrete trial training and mass trials in naturalistic environments, with large N studies, can lead to further research to enhance quality and service and support evidence-based practices and continuous improvement.

Limitations

This replication study has limitations. A non-random sample was used; therefore, there is no ability to generalize to any larger population. Given the nature of this mixed model approach, it was impossible to determine statistically significant differences between the groups relative to discrete trial training, mass trials, and naturalistic environment training.

Peterson, Dodson, Hisey, Sherwin, and Strale [6] and Peterson, Dodson, and Strale [7] emphasized that regarding the seven threats to internal validity, in terms of the impact of history, there may be extraneous variables not part of the study or any external events that may have affected outcomes. Maturation involves age-related bodily changes and includes age-related physical changes that can occur with time, such as hunger, tiredness, fatigue, wound healing, surgery recovery, disease progression, etc. Testing relates to the notion that the test may affect the children’s responses when tested again. These are less of an issue when the tests are routine. Instrumentation refers to any change in measurement ability, including any judge, rater, etc. Statistical regression is the tendency for individuals who score extremely high or low on a measure to score closer to the mean of that variable the next time they are measured on it. Selection refers to the potential bias in selecting participants who will serve in the experimental and control groups. Mortality refers to the differential loss of study participants, drop-out rate, or attrition [6,7].

Given this is a repeated measures scenario using a within-subjects design, each subject serves as its own control. No control group is used as ethical issues preclude withdrawing a treatment intervention for the research subjects. Additionally, as this is a one-month snapshot study, assessing a sample of patients longitudinally or a prospective study for a more extended period will be informative. There appears to be a need in the literature to analyze discrete trial training and naturalistic environment training with repeated measures using large N designs that call for future inquiry [6,7].

## Conclusions

This replication study reaffirmed the efficacy of ABA using discrete trial training and mass trials within a naturalistic environment with autistic individuals during a one-month snapshot. Statistically significant mean differences in target behaviors were determined across the three time points, as well as a statistically significant association was found between time and age within 1-4 years, 5-8 years, and a portion of 9-12 years, but not within the 13-16 years and 17-73 years age groups. The reporting of ongoing intervention outcomes provides further justification for continued treatments relative to target behavior mastery with autistic individuals. This is necessary to confirm prior research results and explain why a study of this nature is necessary and how it contributes to the existing body of literature. Despite efficacy variability in the clinical implementation of ABA due to increasing numbers of individuals diagnosed with ASD, validation of ABA as an evidence-based standard of treatment for ASD with new ongoing data remains necessary.
